# History and Evolution of the Hypervirulent *Clostridioides difficile* Ribotype 027 Lineage

**DOI:** 10.3390/microorganisms13102376

**Published:** 2025-10-15

**Authors:** Isabella A. Tickler, Richard V. Goering, Fred C. Tenover

**Affiliations:** 1 Cepheid, Sunnyvale, CA 94089, USA; 2 Department of Medical Microbiology and Immunology, Creighton University, Omaha, NE 68178, USA; richardgoering@creighton.edu; 3 College of Arts and Sciences, University of Dayton, Dayton, OH 45469, USA; ftenover1@udayton.edu

**Keywords:** *Clostridioides difficile*, glucosylating toxins, binary toxin, ribotyping, pathogenesis

## Abstract

*Clostridioides difficile* was first identified in 1935 and subsequently emerged over the next several decades as the predominant bacterial cause of healthcare-associated gastrointestinal infections, placing a significant burden on healthcare systems worldwide. A major driver of the rapid rise in the incidence of *C. difficile* infection (CDI) was the emergence and spread of a hypervirulent strain, which became known as PCR ribotype 027 (RT027). The *C. difficile* RT027 strain produced not just the typical toxin A and toxin B virulence factors but also expressed a third toxin called binary toxin that enhanced pathogenicity. Interestingly, the *C. difficile* RT027 strain apparently emerged at least twice in geographically distinct areas. The two lineages can be differentiated by their resistance or susceptibility to fluoroquinolones. Other ribotypes of *C. difficile* that also express binary toxin have emerged recently, some of which are highly related genetically to RT027. The aim of this study is to integrate genomic data and published literature to clarify the emergence, divergence, and apparent decline of *C. difficile* RT027.

## 1. Introduction

*Clostridioides difficile* (formerly known as *Clostridium difficile*) is a spore-forming, Gram-positive, anaerobic bacterium that can cause a wide spectrum of gastrointestinal infections in humans, ranging from mild diarrhea to pseudomembranous colitis, to toxic megacolon and death [1]. *C. difficile* was first identified in 1935 by Hall and O’Toole [2], and subsequently emerged, over the next several decades, as the predominant bacterial cause of healthcare-associated gastrointestinal infections, placing a significant burden on healthcare systems worldwide [3,4].

A major driver of the rapid rise in the incidence of *C. difficile* infection (CDI) was the emergence and spread of a hypervirulent strain, which produced a novel virulence factor known as binary toxin in addition to the typical toxin A and toxin B [5]. This strain became known primarily as PCR ribotype 027, but was also identified variably as North American pulsed-field gel electrophoresis (PFGE) type NAP1, and restriction endonuclease analysis (REA) type BI, although there was not total concurrence among the results of the typing methods [6,7]. While other ribotypes of *C. difficile*, such as RT078, also produced binary toxin, it was primarily *C. difficile* RT027 that spread rapidly throughout North America and Europe in the early 2000s, causing severe hospital outbreaks of CDI characterized by high morbidity and mortality [8,9]. Hospital outbreaks caused by *C. difficile* RT027/NAP1/BI were difficult to control, often requiring multiple infection prevention and control interventions [10]. Since most reports of typing studies of *C. difficile* isolates over the last decade have used PCR ribotyping, we will refer to this strain as RT027 in this review.

Approximately a decade after the rapid rise in the incidence of CDI globally, due in part to the spread of the RT027 strain, a gradual decline in its incidence was generally observed in North America and many countries in Europe. This was, in part, related to the implementation of infection control and prevention policies, such as greater emphasis on handwashing, hospital cleaning protocols, and restriction of fluoroquinolone prescriptions [11,12,13]. However, hospitals in Central Europe still reported high incidence rates of RT027 and closely related strains [13]. This time period also saw the rise of several other novel ribotypes that were very similar genetically to RT027, including RT036, RT176, and RT244.

**Figure 1:**
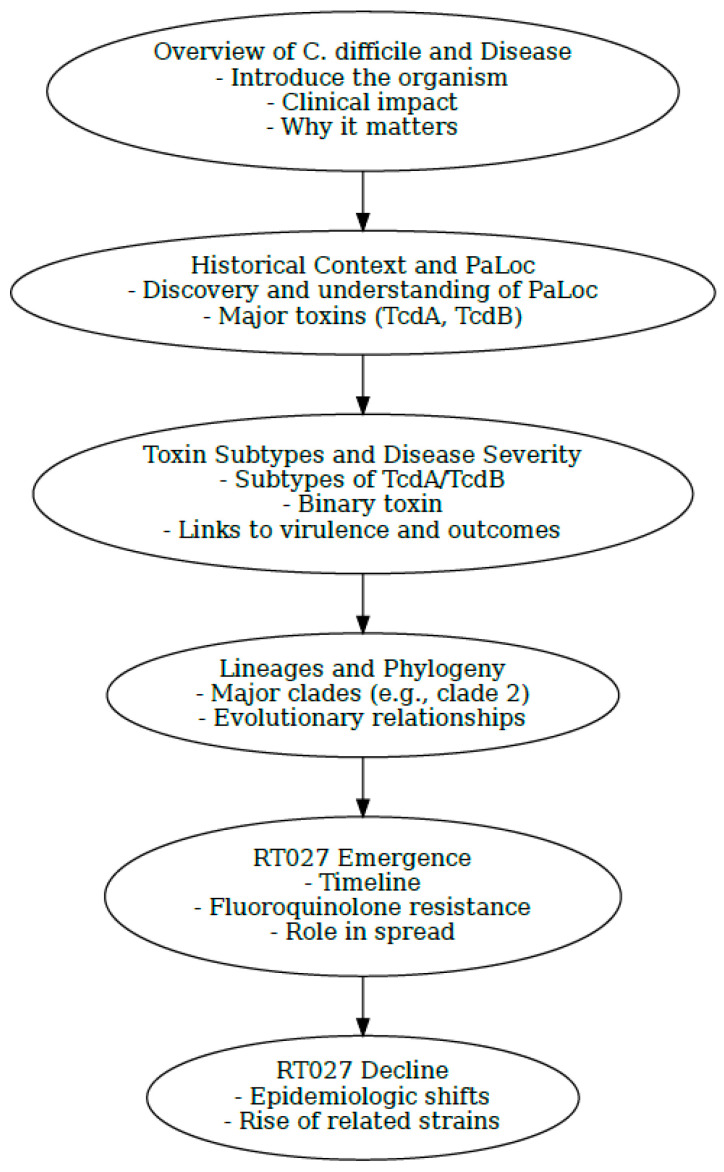
Visual flowchart summarizing key milestones and concepts discussed throughout the review. Flowchart generated by Microsoft Copilot GPT-4, 25 September version (Microsoft 2025, https://copilot.microsoft.com).

This review aims to synthesize genomic data and global literature to better understand the epidemiology and evolution of *Clostridioides difficile* RT027, with particular focus on its rise, divergence, and apparent decline. By integrating whole genome sequencing insights, we highlight how strains virtually indistinguishable from RT027 may have gone undetected under newly assigned ribotypes, obscuring the true dynamics of this lineage. To help navigate this complex history, we provide an introductory flowchart (Figure 1) of the key concepts discussed throughout the review.

## 2. Methods

A total of 28 sequences of *C. difficile* strains previously characterized as belonging to clade 2 were downloaded from public databases EnteroBase (https://enterobase.warwick.ac.uk/) and GenBank (National Center for Biotechnology Information). A list of the strains and their characteristics can be found in Supplementary Table S1. Briefly, the selected dataset comprises 12 distinct RT027 sequences alongside sequences from 8 different multilocus sequence types (MLST), as well as several other ribotypes collected from 11 different countries. The strains were selected to span a broad temporal range, from the pre-epidemic period up to 2019, to include representatives from both FQR1 and FQR2 lineages, with the aim of illustrating their phylogenetic relationships. Sequence analysis included multi-locus sequence typing (MLST carried out using the 7-locus scheme) performed using the CLC Genomics Workbench version 24.0 and CLC Microbial Genomics Module version 24.0 (QIAGEN Bioinformatics, Aarhus, Denmark). A phylogenetic tree of the clade 2 *C. difficile* strains was constructed using CSIPhylogeny with default settings, the single nucleotide polymorphisms (SNPs) analysis tool by the Center for Genomic Epidemiology (https://www.genomicepidemiology.org/services/, accessed on 22 May 2025). The SNP tree was imported with Newick Importer 1.0 and metadata were visualized using CLC Genomics Workbench version 24.0. The sequences of the 28 strains and selected resistance determinants were compared to the RT012 reference strain 630 (Accession CP010905).

Figure 1 (flowchart) and Figure 8 (map of Europe) were created using Microsoft Copilot (Microsoft Corporation, Redmond, WA, USA), an AI-powered assistant based on the GPT-4 architecture. The flowchart was designed to summarize key evolutionary events, while the map was generated to illustrate the geographic distribution of *Clostridioides difficile* ribotype 027 across Europe, based on published literature. Metadata overlays, including country-specific annotations and prevalence indicators, were subsequently added to the map using Adobe Acrobat (Adobe Inc., San Jose, CA, USA).

## 3. A Brief Review of *C. difficile* Disease

The first description of one of the more deadly forms of CDI, i.e., pseudomembranous colitis (PMC), was reported in 1893, long before the antibiotic era began, by Finney, who described the autopsy findings of a patient with severe colitis with what he referred to as a “diphtheritic membrane” in the colon [14]. After the introduction of antibiotics, PMC became more common and the role of antibiotic therapy, especially the use of clindamycin, became associated with the onset of diarrheal disease often with membrane formation in the colon [15]. The disease was commonly referred to as “antibiotic-associated diarrhea”. However, the disease was often mistakenly attributed to infection with *Staphylococcus aureus*, which was frequently recovered in cultures of colonic biopsy specimens taken from symptomatic patients [14]. *C. difficile* was first identified in 1935 in the intestinal tracts of newborns [2]. In fact, *C. difficile* was noted as a frequent colonizer of the intestinal tract of newborns where it reached its highest rate of colonization between 6 and 12 months of age [16]. Importantly, *C. difficile* was not described as the etiologic agent of antibiotic-associated colitis until years later in 1978 by Bartlett et al. and by George et al. [17,18]. Experimental animal models, especially the hamster model, have been used over the years as surrogates of human infection to measure the effect of the toxins produced by *C. difficile* [19]. Initial findings from animal model studies led to the erroneous conclusion that toxin A was the more virulent of the two large clostridial toxins (i.e., toxin A and toxin B). Thus, the initial immunoassays designed for diagnosing CDI only detected toxin A and not toxin B. When later studies indicated the significant role of toxin B in disease, newer immunoassays were designed to detect both toxins to enhance the sensitivity of laboratory testing [20]. Culturing of stool specimens for *C. difficile* followed by testing of the isolate in pure culture for toxin production (i.e., toxigenic culture) was the gold standard for laboratory diagnosis of CDI for many years. This was replaced by toxin testing of the stool directly or by the use of nucleic acid amplification on stool specimens for toxin A or toxin B genes. Screening stool specimens for the presence of the glutamate dehydrogenase (GDH) antigen, which is present on the cell walls of both toxigenic and non-toxigenic isolates of *C. difficile*, followed by testing for toxin production was an alternate strategy [21].

Understanding of the genetics underlying the virulence of *C. difficile* began in 1996 with the publication of the structure of the pathogenicity locus (PaLoc), i.e., the genetic environment surrounding the genes encoding toxins A and B and their regulatory elements. This was critical for distinguishing toxigenic from non-toxigenic strains of *C. difficile* [22].

In 1988, a *C. difficile* strain isolated from a patient with symptoms of PMC was shown to produce an additional toxin to toxins A and B. The new toxin, designated as “binary toxin”, was an ADP-ribosyltransferase that was encoded outside of the PaLoc and was capable of modifying cell actin [23]. This newly described strain harboring the binary toxin was the first historic report of the *C. difficile* RT027 strain.

*C. difficile* is one the leading bacterial causes of healthcare-associated infections and was designated an urgent threat by the Centers for Disease Control and Prevention (CDC) in 2019 [24]. Clinical manifestations range from mild diarrhea to severe complications such as pseudomembranous colitis and toxic megacolon. According to CDC data from 2022, CDI remains a high-burden infection in the United States, with over 116 cases per 100,000 population and an estimated 30,000 deaths annually. In Europe, the European Centre for Disease Prevention and Control (ECDC) reports an incidence of approximately 30 cases per 100,000, with an attributable mortality rate of 5.5%, corresponding to 8382 deaths per year [25,26,27,28].

## 4. The Pathogenicity Locus and Its Relationship to CDI

**Figure 2:**
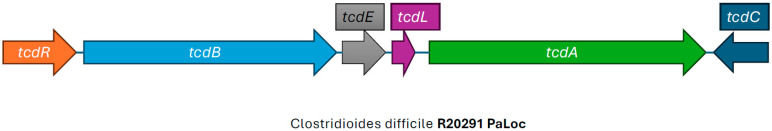
Illustration of the pathogenicity locus, adapted from SnapGene (version 5.1.7) view of the pathogenicity locus of reference *Clostridioides difficile* RT027 strain R20291.

CDI is mediated by multiple virulence factors including the two large clostridial toxins, toxin A and toxin B, more appropriately called enterotoxin TcdA, and the cytotoxin TcdB. These toxins are encoded by the *tcdA* and *tcdB* genes, respectively, found in the PaLoc [29,30] (Figure 2). The PaLoc also contains the *tcdR* gene, which produces an RNA polymerase sigma factor that positively regulates toxin production; *tcdE*, a gene involved in toxin secretion; and *tcdC*, an anti-sigma factor that is thought to negatively regulate toxin production [31]. The mechanism of secretion of the large clostridial toxins is still largely unknown, but likely involves other genes found in the PaLoc that encode holin-like proteins (*tcdE*) and an endolysin remnant (*tcdL*) [32]. Isolates that carry only *tcdB* and not *tcdA* have been described and encode an intact lysin, *cwlH*, downstream of *tcdE,* consistent with the proposed bacteriophage origin of the PaLoc, and with a potentially different mechanism of pathogenesis [32,33,34]. The PaLoc has been reported to be mobilized from toxigenic to non-toxigenic *C. difficile* strains via horizontal gene transfer [35,36]. This is why simply detecting the presence of *C. difficile* in stool specimens, either by anaerobic culture or by detecting the presence of glutamate dehydrogenase (which is present on all strains of *C. difficile*), is not sufficient for diagnosing CDI, since non-toxigenic strains typically do not cause disease.

## 5. The Origin of the PaLoc

A report in 2015 of a case of CDI, which by culture yielded a *C. difficile* isolate that only produced TcdA and was negative for the *tcdB* gene, was surprising. The authors described the isolate as containing a “Mono-Toxin PaLoc” since they confirmed the complete absence of *tcdB*, *tcdC*, and the holin-encoding *tcdE* gene, which was replaced by *uviB,* an alternative holin-like protein [37]. The same study also identified a *C. difficile* strain characterized by a different Mono-Toxin PaLoc, this one containing only *tcdB, tcdR,* and *tcdE.* Both Mono-Toxin PaLoc strains (TcdA only and TcdB only) belong to a cryptic clade of *C. difficile* designated as C-I. The presence of two “prototype” PaLocs, each containing only a single toxin gene, either *tcdA* or *tcdB*, suggests that in the past, the two toxin genes and their associated genetic loci existed in separate genetic elements in distinct *C. difficile* strains. It further suggests that there was a fusion event between the two prototypical Mono-PaLocs that generated the current PaLoc structure that contains both *tcdA*, *tcdB*, and the respective loci that regulate gene expression and facilitate secretion [37]. Because the Mono-Toxin PaLocs have been identified only in a cryptic clade, it is conceivable that *C. difficile* became a much more successful pathogen only after the merging of the two PaLocs, which is what is seen in the much more successful clades 1–5 [38,39].

## 6. The Large Clostridial Glucosylating Toxins: TcdA and TcdB

**Figure 3:**
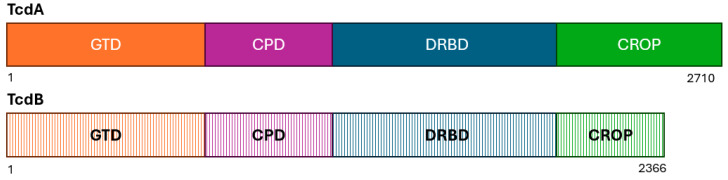
Schematic diagram showing the four domains of TcdA and TcdB: the glucosyltransferase domain (GTD; orange), the cysteine protease domain (CPD; pink), the delivery and receptor-binding domain (DRBD; blue), and the combined repetitive oligopeptides (CROPs). The stippled colors of TcdB indicate the greater sequence variable of these domains compared to those of TcdA. Adapted from references [40,49].

Both toxins TcdA and TcdB share a similar multi-domain structure (Figure 3) and catalyze the glucosylation of small Rho proteins affecting several cellular functions that ultimately damage the cells of the intestinal tract [30,40]. The roles of the two toxins in virulence, however, are very different [41].

TcdA is an enterotoxin comprised 2710 amino acids that was originally thought to be the main virulence factor in CDI, due to studies of the effect of purified toxin A in the hamster model, coupled with the lack of damage produced by purified toxin B when used in the rabbit and mouse models. However, these misconceptions were soon dispelled through additional experimental data indicating a key role of TcdB [38,42,43,44]. The choice of the hamster as a model of human infection was unfortunate. In fact, both TcdA and TcdB contribute to the pathogenesis of CDI in humans. However, there have been reports of severe CDI caused by strains lacking an intact *tcdA* gene and therefore producing only TcdB [45]. Furthermore, antibodies to TcdB and not TcdA blocked disease in more recent animal experiments, further pointing to the major role of TcdB in CDI [46]. The monoclonal antibody bezlotoxumab used for therapy of CDI is based on antibodies directed to TcdB [47]. Although antibodies targeting TcdB are more critical for blocking the pathogenicity of CDI in humans than antibodies against TcdA, both toxins can act synergistically to cause significant damage to the intestinal lining.

The exact role of TcdA in CDI is still being debated [29]. It is presumed that TcdA targets different membrane receptors than TcdB and appears to affect fewer cell types [37,48]. Cytotoxin TcdB consists of 2366 amino acids, can affect all cell types in the gut, and cause cell death by a variety of mechanisms. This includes necrosis of cells at high toxin concentrations, which is aided by high levels of reactive oxygen species (ROS); pyknotic cell death, which is mediated by glucosyltransferase and ROS; and pyroptosis, which occurs through the induction of a potent inflammatory response and pore formation in cellular membranes [38,48].

## 7. Variants of TcdA and TcdB and Their Relationship to RT027

The sequence of TcdA is highly conserved, with a maximum amino acid diversity of only 2.25%. While seven subtypes of TcdA have been identified, TcdA receptors remain relatively conserved [50]. By comparison, the sequence of TcdB shows considerable variability with a maximum residue difference of 15.13%. There have been 12 TcdB variants identified; however, variants 1–4 are the most common and are present in over 99.6% of pathogenic *C. difficile* isolates. This is consistent with the accelerated evolution of the *tcdB* gene [51,52]. Many of the differences in the amino acids observed in TcdB are related to receptor variability. The TcdB variants appear to also manifest differences in disease progression and the ability to adapt to a variety of hosts [50,51,52]. For example, in a mouse model, TcdB1 produces more severe symptoms, while TcdB2 causes stronger edema, and TcdB3 causes more severe cell infiltration [52]. The eight main TcdB subtypes, TcdB1 through TcdB8, loosely align with the *C. difficile* phylogeny [51,53]. TcdB1, the prototypic toxin, which continues to be the most common toxin variant observed, is mostly associated with clade 1 strains, which is also the most common clade causing human infections. Clade 1 includes the successful ribotypes RT001, RT002, RT014, RT018, and RT106 [53]. TcdB1, TcdB2, and TcdB3 tend to mediate more severe CDI symptoms than the other toxin subtypes. TcdB2 in particular is the subtype produced by several MLST types within clade 2, including ST1 to which RT027, RT176, RT181, and RT198 belong. Thus, RT027 is associated with one of the more potent TcdB variants, which is consistent with the severity of RT027 infections [51]. TcdB3 is also associated with epidemic *C. difficile* strains, specifically RT017, which is a hypervirulent strain prevalent in East Asia [52]. TcdB4 likely emerged as a result of recombination among TcdB2, TcdB3, and TcdB7 and tends to be a less potent toxin subtype and less virulent in the hamster model [51]. RT244 (ST41) and RT019 (ST67) may produce either TcdB2 (typical of ST1/RT027) or the TcdB4 toxin subtype. This variable TcdB production is consistent with RT244’s characteristics as an outlier strain within clade 2 [54]. RT036 (ST62) produces TcdB4. Variants of the TcdB toxin have been linked to differing levels of disease severity, and several, particularly those produced by *C. difficile* isolates from cryptic clades, pose challenges for detection using antibody-based diagnostic tests [34,52]. Moreover, *C. difficile* isolates belonging to cryptic clades may carry highly divergent *tcdA* or *tcdB* genes, which can complicate detection by PCR-based assays targeting the major toxin genes [55].

## 8. Binary Toxin—The *C. difficile* Transferase (cdt) Locus

**Figure 4:**
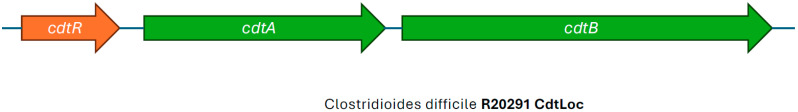
Illustration of the CDT Locus of *Clostridioides difficile* RT027 strain R20291, showing intact cdtA and cdtB genes, adapted from view developed in SnapGene (Version 5.1.7).

*C. difficile* RT027 and a limited number of strains with other ribotypes produce binary toxin in addition to TcdA and TcdB. Binary toxin is more formally known as C. *difficile* transferase (CDT). It is a two-component toxin encoded by the genes *cdtA* and *cdtB,* which are located in a chromosomal region outside of the PaLoc called the CDT locus (Figure 4) [56,57]. CDT is formed by two molecules that are not linked to each other; however, both are needed for toxin activity [58]. Binary toxin is secreted by two protein components (CDTa and CDTb), where the CDTb component forms a pore and helps translocate the CDTa component through the host cell [59]. Recent reports have described rare cases of CDI caused by *C. difficile* strains that only produce CDT but are negative for TcdA and TcdB [60,61]. The role of CDT in CDI pathogenesis is still not completely understood; however, its toxicity and effect on the actin cytoskeleton of host cells have been confirmed in vitro [56,62]. Most *C. difficile* strains, like RT027, that produce CDT do so in addition to TcdA and TcdB. The production of CDT has been associated with more severe CDI symptoms and a higher recurrence rate, especially when the strain harbors TcdB [63,64,65].

## 9. The Ambiguities of Traditional *C. difficile* Typing Methods

The epidemiology of CDI in healthcare systems has traditionally relied on a number of different strain-typing methods to differentiate sporadic cases of CDI from outbreaks of disease. During the early 2000s, several different methods were used to type *C. difficile* strains, including polymerase chain reaction (PCR) ribotyping, in which the 16S-23S intergenic spacer regions were amplified using PCR resulting in several DNA fragments, which were then separated by polyacrylamide gel or capillary gel electrophoresis; pulsed-field gel electrophoresis (PFGE), which used infrequent cutting restriction enzymes to digest the bacterial genomic DNA into large pieces followed by the separation of the fragments using contour clamped gel electrophoresis; restriction endonuclease analysis (REA) where the bacterial genome was digested with frequent cutting endonucleases followed by agarose gel electrophoresis, which produced complex fragment patterns; multilocus sequence typing (MLST), which involved PCR amplification of genomic DNA and sequencing of a limited number of housekeeping genes; toxinotyping, which analyzes where polymorphisms of the PaLoc were amplified by PCR and identified via gel electrophoresis; and multilocus variable-number tandem-repeat analysis (MLVA), where variations in tandem repeats within genetic loci were identified via PCR and the fragment patterns were delineated by gel electrophoresis [7]. More recently, whole-genome sequencing (WGS), especially using the application of next-generation sequencing (NGS) methods, has enabled analysis of large DNA sequence datasets providing high-resolution typing and phylogenetic relationships [6,7,66,67]. Of these methods, PCR ribotyping was the most broadly used globally, followed by PFGE, which was mostly carried out in public health laboratories in the United States, and REA typing, which was performed in a single laboratory in the United States. Considerable effort went into trying to harmonize PCR ribotyping among laboratories to make the data more consistent and portable from country to country. Nonetheless, PCR ribotyping had drawbacks, such as the inability to differentiate strain type RT020 from RT014, and types RT078 from RT126 in many laboratories. The limited availability of ribotype control strains, especially for newly described ribotypes, was also an issue for many laboratories globally. The introduction of capillary gel electrophoresis-based PCR ribotyping and online databases for quick and accurate analysis mitigated some of these limitations, although comparing distances and numbers of ribosomal interspace regions rather than nucleotide base sequences limits the former method’s discriminatory power and specificity [68]. This led to the adoption of WGS-based typing methods, which were more reproducible and portable. WGS data were critical for showing the evolution of RT027 strains and highlighting the emergence of novel ribotypes highly related to RT027 [68,69].

## 10. *C. difficile* Lineages and Phylogeny

WGS typing has illuminated the complex nature of the *C. difficile* phylogeny much more clearly than earlier typing methods, including ribotyping [70]. Although *C. difficile* evolved as a genetically diverse species millions of years ago, it only appeared as a pathogen relatively recently and outbreaks caused by epidemic strains have been identified only less than three decades ago [71]. There are five major lineages of *C. difficile* that have been identified through multilocus sequence typing (MLST) as distinct clades. These are designated as clades 1 through 5. In addition, there are “cryptic clades”, designated as C-I through C-V. The cryptic clades typically contain non-toxigenic *C. difficile* strains or strains with divergent toxin genes that are more likely to be detected in specimens from the environment rather than from human infections [72].

Acquisition of the PaLoc sequences associated with toxin production by formerly non-toxigenic *C. difficile* strains likely occurred multiple times among the various *C. difficile* clades transforming them into virulent pathogens [73]. Clade 1 is the most widely distributed of the *C. difficile* lineages globally, contains over 200 sequence types (ST), and is the most diverse genetically. This clade may have been the first to acquire a PaLoc and is comprised of both toxigenic and non-toxigenic strains. Clade 2 contains several strains that have been reported as hypervirulent, such as those belonging to ST1. Clade 3 contains highly related strains, including RT023, a CDT-positive strain associated with severe CDI symptoms [74]. Clade 4 includes RT017 (ST37), which is a hypervirulent strain predominant in East Asia and known for its TcdA−/TcdB+ phenotype [73]. Clade 5 is the most evolutionarily distant branch of the *C. difficile* phylogeny and includes ribotypes RT033, RT078, and RT127, which belong to ST11, which are well established in livestock and agriculture, but also successful strains among humans [75]. According to Knight and colleagues, fluoroquinolone resistance may have also been the driver of the successful global expansion of RT078/126, which is resistant to fluoroquinolones in isolates of human clinical origins, but susceptible in isolates from livestock [75].

## 11. The Emergence of *C. difficile* RT027

The earliest report of what was later identified as *C. difficile* RT027 dates back to 1985 when *C. difficile* strain CD196, characterized by the production of the newly described CDT in addition to TcdA and TcdB, was isolated from a patient with CDI in Paris, France [23]. The next reported isolation of a *C. difficile* RT027 strain was not until 1988. The organism, *C. difficile* strain 1675, was cultured from a patient with CDI in a hospital in Minnesota in the United States [76]. The early and sporadic RT027 strains were not associated with outbreaks and were considered, like all other strains causing CDI, to be a complication of antimicrobial therapy. Once protocols for typing of *C. difficile* using ribotyping, PFGE, and REA were introduced a few years later, these historical strains, which were available in isolate collections, were typed and assigned to the genotype RT027/NAP1/BI [77,78]. The lack of recognition of the outbreak potential of RT027, along with the lack of effective infection prevention activities, led CDI cases in the United States to increase from 2.7 per 10,000 hospital admissions in 1987 to 4.2/10,000 in 2001, and from 25,000 cases in the year 2000 to 54,000 cases in 2003, a 46% increase [79,80,81]. When a convenience sample of *C. difficile* isolates from outbreaks of CDI occurred between 2000 and 2003 in eight US hospitals were characterized, 51% of the strains were classified as RT027/NAP1/BI, and, unlike their historic counterparts, were resistant to fluoroquinolones [5]. A hospital in Quebec, Canada reported an increase in CDI cases from 102 cases per 100,000 individuals in 1991–92 (pre RT027 emergence) to 866 cases per 100,000 individuals in 2003 (post RT027 emergence) [8]. In Calgary, Canada prior to the year 2000, hospitals reported 2–4 cases of CDI per 500 bed/months, but between 2000 and 2001, the numbers increased to 18–22 per 500 bed/months [82]. Typing of isolates collected in Canada between 2000 and 2004 showed that RT027 represented 10.7% of all isolates obtained from the Calgary Health Region and 75.2% of isolates from the Montreal area (for the 2003–2004 period) [83]. Similarly, in the United Kingdom, a 2005 report by the Health Protection Agency’s Communicable Disease Surveillance Centre (CDSC) reported a 98% rise in *C. difficile* cases between 2001 and 2004 [84]. As of 2008 in England, 42% of 2084 *C. difficile* isolates collected were RT027 [85]. A comprehensive phylogenetic and evolutionary analysis by He et al. showed that *C. difficile* RT027 underwent a major population expansion right around the time these outbreaks were first reported. Although a number of reviews have been focused on this epidemic strain, not much is known about which factors drove such a successful global expansion [71]. However, it appears that the acquisition of fluoroquinolone resistance through a Thr82Ile mutation in the *gyrA* gene may have contributed to the emergence and expansion of the epidemic *C. difficile* RT027 strain.

A study by Spigaglia and coworkers demonstrated that both moxifloxacin (MXF) and levofloxacin (LEV) can select for *gyrA* mutants in *C. difficile* in vitro, which mediate fluoroquinolone resistance [86]. They also found that MXF does not reach inhibitory concentrations in the intestine in the early stages of treatment, allowing subpopulations of *C. difficile* to survive using other resistance mechanisms, such as efflux pumps, to multiply to large numbers, and to acquire stable *gyrA* mutations [86]. Furthermore, the Thr82Ile mutation in *gyrA* does not have a detectable fitness cost to the organism, so it can be maintained even in the absence of antibiotic pressure [87,88]. The introduction of the more potent fluoroquinolones, i.e., moxifloxacin and gatifloxacin, in the United States and Europe (particularly in the United Kingdom) in 1999 may have been instrumental in the expansion of the RT027 strain. In the United States, a study tracking fluoroquinolone use in urinary tract infections between January 2000 and March 2020 reported peak levels of use in January 2004 (69.8% of encounters) and a subsequent decline following several warnings of potential adverse events, including the 2008 FDA black box warning, for the risk of tendinitis and tendon rupture. This was followed in 2018 by further restrictions from the European Medicines Agency, due to side effects observed with fluoroquinolone prescriptions [89,90]. The chronological trend in fluoroquinolone usage coincides with the expansion and apparent decline of the *C. difficile* RT027 epidemic strain observed in the United States (Table 1). A hospital and affiliated long-term care facility in Cleveland, OH (USA) reported that a reduction in fluoroquinolone prescriptions for inpatients between 2009 and 2018 coincided with a decline in percent of *C. difficile* RT027 isolated from 70% to 10%, plus a significant decline in healthcare-associated CDI [91]. The study also reported that RT027 strains were replaced by a variety of strain types, rather than a predominant ribotype, including RT014/020 and RT078/126. Another hospital in Illinois (USA) compared the rate of first-episode CDI cases between 2005 and 2007 and 2013 and 2015 and reported a decrease in the prevalence of RT027 from 72% to 19%. This change coincided with a 50% reduction in fluoroquinolone usage between the two periods, in particular with the cessation of gatifloxacin prescriptions by 2007 and a significant reduction of moxifloxacin prescriptions by 2015 [92]. This study also noted a significant increase in prevalence of other ribotypes, especially RT014/020 and RT106. Similarly, in the United Kingdom, there was a steep rise in the incidence of CDI from 1998 to 2006, which coincided with the peak prescribing of fluoroquinolones, followed by a decline in CDI cases, which coincided with the restriction of fluoroquinolone prescriptions in 2007 [93]. In Oxfordshire, UK, CDI caused by fluoroquinolone-resistant *C. difficile* strains declined from 67% in 2006 to 3% in 2013, showing the profound impact of restricting fluoroquinolone use, compared to other infection control measures [93]. This supports the hypothesis that the rise and apparent fall of cases of CDI caused by RT027 may have been related at least in part to changing antibiotic usage patterns.

The *C. difficile* RT027 strain type apparently acquired FQR mutations twice in two independent genetic events giving rise to two distinct RT027 lineages [94]. The first lineage was designated FQR1 and was initially reported in a *C. difficile* RT027 isolate recovered from a patient in Pennsylvania, USA in 2001. This lineage was ultimately associated with healthcare-associated outbreaks of CDI in the United States, South Korea, and Switzerland. The second lineage, known as FQR2, also originated in North America but then spread rapidly to Europe with multiple introductions into the United Kingdom and to Australia [94]. Given the widespread use of fluoroquinolones in healthcare in the early 2000s [95], the acquisition of fluoroquinolone resistance by *C. difficile* RT027 strains was likely a significant driving force for its global spread.

It is also possible that metronidazole use, in lieu of vancomycin and fidaxomicin, continued to select for more resistant strains like RT027 and the closely related RT198 and RT955 strains, the latter of which has recently been associated with an outbreak in the United Kingdom [96,97]. Metronidazole is no longer recommended as a first-line agent for CDI treatment, when alternatives like fidaxomicin or vancomycin are available; however, it is still widely used in some regions of Europe due to its lower cost and wide availability [98,99,100]. Another potential selective advantage for RT027 may have been the development of rifampicin resistance, which is especially high in RT027 and closely related strains [101,102,103]. Rifampicin is frequently used in orthopedic surgery cases and in the treatment of *Mycobacterium tuberculosis* infections, but it can also promote CDI with rifampicin-resistant strains like RT017, RT027, RT176, and RT001, all of which are associated with higher virulence and outbreaks [104,105].

**Table 1:** Timeline of significant events related to the emergence and spread of *C. difficile* RT027.

Year	Location	Event	Type of Change	References
1999	United States and Europe	Introduction of moxifloxacin (4th gen fluoroquinolone)	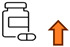	[90]
2000–2003	United States	Increase in CDI cases from 25,000 in year 2000 to 54,000 in year 2003	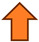	[80]
2003	Canada	Increase in CDI cases from 102 per 100,000 patients in 1991–92 to 866 per 100,000 patients in 2003	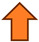	[8]
2001–2004	United Kingdom	A 98% rise in CDI cases	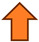	[84]
2006	United States and United Kingdom	Peak fluoroquinolone use	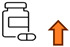	[90,93]
2007	United Kingdom	Restriction of fluoroquinolone prescriptions	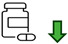	[93]
2009–2011	United States and Europe (part)	Decline in CDI cases caused by RT027	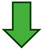	[101,106,107,108]
2016–2017	Europe	RT027 was the third frequent ribotype overall in Europe, except for Czech Republic, Hungary, Poland, and Slovakia	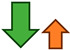	[28]
2018–2020	Europe	ECDC survey: further decline of RT027 (now 11th most frequently reported)	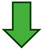	[28]
2020	Greece	Outbreak of CDI due to a 027-like PCR ribotype 181	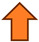	[109]
2024	Greece	RT181 is the predominant ribotype in a rehabilitation center (76.6%)	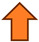	[110]
2024	Poland	Study reported RT027 (77.8%) still dominant ribotype, emergence of RT955 (12.7%)	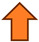	[111]

## 12. The Potential Impact of Other Antimicrobial Resistance Determinants

*C. difficile* has a highly plastic genome, characterized by a large number of mobile genetic elements (MGEs), often constituting as much as 11% of the genome, which can carry specific antimicrobial resistance determinants [112]. Genes carried in MGEs can confer resistance to aminoglycosides, tetracycline, macrolides, and beta-lactams. Horizontal transfer of MGEs into *C. difficile* RT027, such as the conjugative transposon Tn*6192*, which was not identified in pre-epidemic RT027 strains, provided a mechanism for further acquisition of antimicrobial resistance and virulence determinants by *C. difficile*. While these resistance determinants did not impact therapy for CDI, since these drugs were not used clinically for treatment, they may have given RT027 strains a greater selective advantage for survival in the human gut when the resident microbiome was dramatically reduced by the presence of antimicrobial agents. This further facilitated the spread and persistence of the RT027 strain [94].

Acquisition of mutations in the *rpoB* gene by certain strains of *C. difficile* confers resistance to rifampicin [113]. A previously described mutation associated with a high level of rifampicin resistance is R505K, while H502N has been associated more with intermediate resistance [114]. R505K was found in 5 of the 12 RT027 strains in our representative sample, once together with H502N. Both RT176 in our convenience sample harbored R505K and H502N, while RT955 had both R505K and V134I mutations, as previously reported [115]. None of the clade 2 strains analyzed in this study harbored the rare pCD-METRO plasmid, associated with metronidazole resistance; however, all strains, with the exception of sequence types other than ST-1 and the pre-epidemic RT027, harbored the T to G mutation in the -10 promoter of the *nimB* gene, which is linked to heme-dependent metronidazole resistance in *C. difficile* and it has been reported to co-occur, as observed in our study, with the Thr82Ile mutation in *gyrA* [116]. An additional mutation, an L155I, was observed in *nimB*, and it was found to be unique to the FQR2 lineage of ST1 strains. The effect of this mutation is still unknown but it seems to be present in strain types that have been reported to have reduced susceptibility to metronidazole, such as RT027, RT176, RT955, and RT181 [117,118]. These mutations associated with rifampicin and metronidazole resistance may have contributed to the regional adaptation of certain ribotypes.

## 13. Did Traditional Typing Methods Obscure Epidemiologic Changes in *C. difficile* Strains?

**Figure 5:**
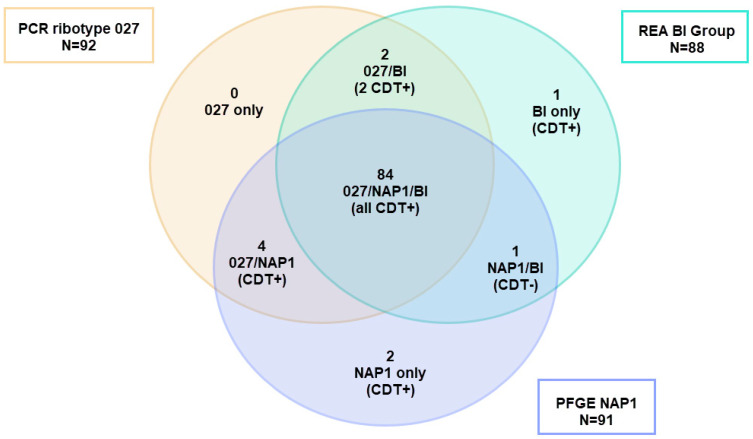
Adaptation of the Venn diagram from reference [6]. Of the 92 isolates typed using three different methods (PCR ribotyping, REA, and PFGE) and identified as PCR ribotype 027, only 89 belonged to PFGE type NAP1 and 84 were BI by REA showing a cluster of 84 strains clustering as 027/NAP1/BI. Two isolates were excluded from the analysis because they were not typed by all methods. REA = restriction endonuclease analysis. PFGE = pulsed-field gel electrophoresis.

**Figure 6:**
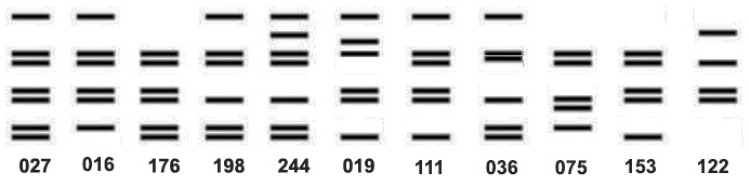
Diagrammatic representation of the ribotype banding patterns adapted from Valiente et al. and Knetsch et al. [120,123] showing the close relationship among the ribotypes.

As noted above, PCR ribotyping and PFGE have been widely used as *C. difficile* strain typing methods to follow the epidemiology of CDI [119]. However, these methods have several limitations including the limited concordance of results among laboratories and the portability of data, i.e., the sharing of information among laboratories. For example, a 2011 study that correlated the typing data for 92 isolates of *C. difficile* showed that the results of three typing methods (ribotyping, PFGE, and REA) significantly overlapped for isolates identified as either RT027, PFGE NAP1, or REA BI, but were not totally concordant. There were outliers that showed unique strain types by each method (Figure 5) [6]. Thus, not all the isolates that were typed as NAP1 by PFGE were RT027, nor were they all REA BI, but they were all very highly related genetically, all produced binary toxin, and all had a deletion at nucleotide 117 in *tcdC*. The same dynamic nature of the RT027 genome that gave rise to FQR1 and FQR2 may be responsible for novel ribotyping bands that produced new PCR ribotypes that were very closely related to prototypical RT027 isolates, including RT176, RT198, and RT244 [120,121,122]. These subtle differences, similar in some ways to antigenic drift, may have contributed to what has been interpreted as the decreasing prevalence of RT027 (Figure 6).

## 14. Clade 2: *C. difficile* Strains Genetically Related to RT027

*C. difficile* RT027 strains, which are members of clade 2, have undergone a marked temporal and geographic evolution. A 2009 study comparing the nucleotide sequences of the historic *C. difficile* CD196 strain isolated in 1985 to an epidemic isolate of RT027 from 2006 found that numerous changes had accumulated in the genome over the years that affected key virulence and survival mechanisms, including motility, toxicity, and antimicrobial resistance. This included mutations associated with resistance to fluoroquinolones [124]. A subsequent study in 2012 compared RT027 strains from the United States and United Kingdom, demonstrating not only different motility and antimicrobial resistance profiles, but also diverging evolution, perhaps further driven by mobilization and exchanges of large chromosomal regions, from which other closely related ribotypes, such as RT176, RT198, and RT244, emerged [121]. It has been hypothesized that the divergent evolution may be in response to changing regional antimicrobial prescribing practices and the repeated transcontinental introductions of RT027 from North America to Europe, the United Kingdom, and Australia [121,125]. Since then, several outbreaks implicating novel clade 2 strains closely related to RT027 have been observed. This includes RT176 reported in Czech Republic, Slovakia, and Poland; RT198 found in Hungary and the Netherlands; RT244 identified in Australia; RT955, which was responsible for a recent outbreak in the United Kingdom and is also present in Serbia and Poland; RT885 from Brazil; and a cluster of RT181 reported in Greece [109,126,127,128,129,130,131,132]. The outbreak of *C. difficile* RT181 was studied by Baktash et al. using sequence-based typing methods, SNP analysis, and PCR ribotyping. They observed a broad range of SNPs within the outbreak strains, probably due to multiple introductions of different RT181 strains (similar to what happened at the beginning of the RT027 outbreak). These subtle changes were more easily detected by whole genome multi-locus sequence typing (wgMLST) than by other typing methods [68]. Most of these strains were either associated with severe disease outbreaks or believed to have RT027-like virulence potential [126,127,128,130,131,132]. Thus, the possibility that some of the apparent decline in the incidence of *C. difficile* RT027 is due to its divergence into closely related strains that are identified as different genotypes by conventional typing methods needs to be considered.

## 15. Single Nucleotide Polymorphism Sequence Data and wgMLST

**Figure 7:**
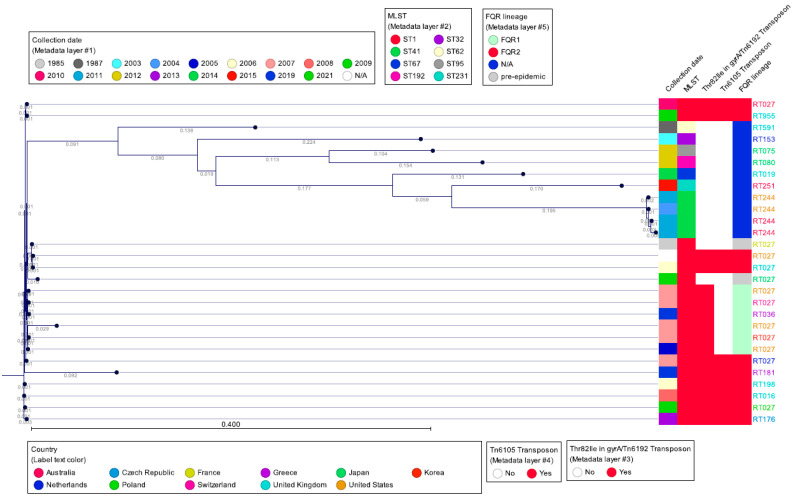
SNP tree of representative PCR ribotypes belonging to clade 2 (reference: *C. difficile* CD630). The SNP tree was created using the Center for Genomic Epidemiology CSI Phylogeny 1.4 tool (https://cge.food.dtu.dk/services/CSIPhylogeny/) with default parameters, and analyzed with CLC Genomic Workbench 24.0.

Figure 7 shows a single-nucleotide polymorphism (SNP) analysis of selected *C. difficile* clade 2 sequences, using the CGE CSI Phylogeny tool, using *C. difficile* strain CD630 as reference. The SNP tree was overlaid with metadata that included geographic origin, cgMLST, the presence/absence of deletion at nucleotide 117 in *tcdC*, and PCR ribotype. The SNP tree shows the high degree of similarity by WGS among RT027, RT016, RT036, RT176, and RT955 strain types, all of which produce binary toxin and have a unique deletion in *tcdC* at nucleotide 117. On the other hand, RT019, RT036, RT075, RT080, and RT251 show less sequence similarity to RT027, yet all produce binary toxin and several, but not all, carry the nucleotide 117 deletion in *tcdC*. The most divergent, although still highly related strains, are those of RT244, which also carry binary toxin and have the nucleotide 117 deletion. It is important to compare the MLSTs of all of these ribotypes as it also indicates the interrelatedness of this group of *C. difficile* strains. MLST type 1 (ST1) to which RT027 belongs also includes RT176, RT955, RT181, RT198, and RT016. However, these ribotypes also are present in other MLST types. Most of the ST1 strains carry the Thr82Ile mutation in *gyrA*, with the exception of strain CD196 and an RT027 strain from Japan, both of which are also negative for Tn*6192* and Tn*6105*, consistent with pre-epidemic strains. The more divergent group of non-ST1 clade 2 strains belongs to various sequence types but lacks the *gyrA* mutation and Tn*6192*/Tn*6105* of the epidemic RT027 and related strains (Figure 7).

## 16. The Decline of RT027; Epidemiological Data

**Figure 8:**
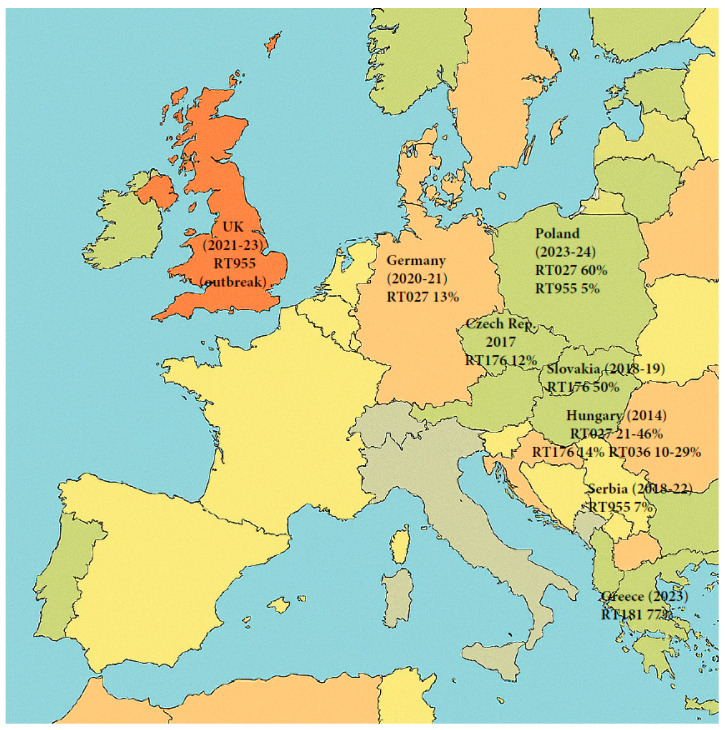
Map of Europe showing the distribution of RT027 and closely related strains according to recent published studies. Map generated by Microsoft Copilot GPT-4, 25 September version (Microsoft 2025, https://copilot.microsoft.com).

During the period of 2009–2011, the United States and several countries in Europe, including the Netherlands and the United Kingdom, started to report a decline in the incidence of CDI caused specifically by strain RT027 [101,106,107,108]. However, other European countries continued to experience outbreaks linked to this strain. According to a 2016–2017 report by the European Centre for Disease Control (ECDC), RT027 was the third most frequently reported ribotype overall, after RT014/020 and RT002, in Europe, although in the Czech Republic, Hungary, Poland, and Slovakia, RT027 and the related clade 2 ST1 strain RT176 were still the most prevalent [28]. A 2016 study conducted in Hungary, in which they typed strains collected from two geographical different regions, found regional variants of RT027: in Budapest, RT027 was the predominant type, followed by the closely related RT176, and then RT036, while in Szeged RT036 was the dominant ribotype, followed by RT027, with complete absence of RT176. The shifting of dominant strains was also noticeable over time, but the authors caution that some of the differences could be an artifact of the ribotype libraries used in the various studies [133]. A 2018–2020 ECDC survey reports a further decline of RT027 (now 11th most frequently reported), but the countries contributing data varied from the earlier report, so a direct comparison with the earlier report is not possible [134]. Between 2018 and 2019, RT176 was the predominant ribotype in Slovakia, representing 50% of all cases in 14 hospitals [135]. A multicenter study conducted across 22 hospitals in the Berlin-Brandenburg region of Germany between 2020 and 2021 identified RT027 to be the second most prevalent ribotype after RT014 [136]. A study conducted by Szarek et al. in 2024 [111] identified RT027 as the dominant *C. difficile* ribotype in the Silesian region of Southern Poland, accounting for 77.8% of cases. Notably, the emergence of new ribotypes such as RT955 (12.7%) was also reported [111]. In a follow-up investigation published in 2025, the same research group confirmed that RT027 remains the predominant strain (60%), with RT955 still present (6%). Alarmingly, metronidazole resistance was detected in 4% of isolates, yet metronidazole monotherapy was administered in 10 out of 130 cases [137] (Figure 8).

## 17. Summary

The decline in the incidence of RT027 cases, at least in some countries, coincides with the emergence of several closely related clade 2 strains with slightly different PCR ribotyping banding patterns, most likely modulated by mutations and rearrangements in response to varying antimicrobial prescribing practices, as demonstrated by the slightly divergent resistance mechanisms observed within FQR lineages of ST1 strains. The multiple introductions of the FQR2 lineage in central Europe, coupled with the different antimicrobial prescription practices, created hotspots of RT027-like strains, as seen in Poland, Slovakia, Czech Republic, all the way down to Greece. This supports the hypothesis that the RT027 problem has not disappeared but has fragmented into a mosaic of clade 2 ribotypes with similar virulence; genomic surveillance will be essential for future CDI control.

## Data Availability

The original contributions presented in this study are included in the article/Supplementary Materials. Further inquiries can be directed to the corresponding author.

## Supplementary Materials

The following supporting information can be downloaded at: https://www.mdpi.com/article/10.3390/microorganisms13102376/s1, Table S1: Characteristics of clade 2 strains visualized in Figure 7.

## Abbreviations

The following abbreviations are used in this manuscript:

CDI	*C. difficile* infection
WGS	Whole genome sequencing
NGS	Next-generation sequencing
PCR	Polymerase chain reaction
PFGE	Pulsed-field gel electrophoresis
REA	Restriction endonuclease analysis
MLVA	Multilocus variable-number tandem-repeat analysis
PaLoc	Pathogenicity locus
CDT	*C. difficile* transferase
MGE	Mobile genetic elements
SNP	Single-nucleotide polymorphism
